# Effect of the preoperative physical status on postoperative nausea and vomiting risk: a matched cohort study

**DOI:** 10.1186/s13741-022-00264-1

**Published:** 2022-09-06

**Authors:** Jong Ho Kim, Haewon Kim, Kookhyun Yoo, Sung Mi Hwang, So Young Lim, Jae Jun Lee, Young Suk Kwon

**Affiliations:** 1 Department of Anesthesiology and Pain Medicine, Hallym University Chuncheon Sacred Heart Hospital, Hallym University Medical Center, 77 Sakju-ro, Chuncheon, 24253 South Korea; 2 Institute of New Frontier Research Team, Hallym University, Chuncheon, South Korea; 3 Department of Anesthesiology and Pain Medicine, College of Medicine, Kangwon National University, Chuncheon, South Korea

**Keywords:** Postoperative nausea and vomiting, Preoperative physical status, Propensity score matching, American society of anesthesiologists physical status classification system

## Abstract

**Background:**

The American Society of Anesthesiologists Physical Status Classification System is commonly used for preoperative assessment. Patient physical status before surgery can play an important role in postoperative nausea and vomiting. However, the relationship between the physical status classification and postoperative nausea and vomiting has not been well defined.

**Methods:**

Adults aged ≥ 18 years who underwent procedures under anesthesia between 2015 and 2020 were included in the study. We analyzed the relationship of postoperative nausea and vomiting with physical status classification score using propensity score matching and Cox hazard regression. Differences in intraoperative use of vasopressor and inotropes and invasive monitoring were investigated according to the classification.

**Results:**

A total of 163,500 patients were included in the study. After matching, classification 1 versus 2 included 43,400 patients; 1 versus ≤ 3, 13,287 patients; 2 versus ≤ 3, 23,530 patients (absolute standardized difference, 0–0.06). Patients with physical status classification ≤ 3 had a significantly lower postoperative nausea and vomiting risk than those with classification 1–2 (physical status classification 1 vs. ≤ 3, hazard ratio 0.76 [0.71–0.82], *P* < 0.001; 2 versus ≤ 3, hazard ratio 0.86 [0.82–0.91], *P* < 0.001). Intraoperative use of vasopressor or inotrope and invasive monitoring were noted more in the high physical status classification than the low physical status classification (absolute standardized difference [0.19–1.25]).

**Conclusion:**

There were differences in intraoperative invasive monitoring and use of vasopressor or inotrope among the classifications, and a score of 3 or higher reduced the risk of postoperative nausea and vomiting more than a score of 1–2.

**Supplementary Information:**

The online version contains supplementary material available at 10.1186/s13741-022-00264-1.

## Background

Postoperative nausea and vomiting (PONV) is a common complication of general anesthesia, occurring in 30 to 40% of patients. In particular, it can occur after outpatient surgery within 24 h of uneventful discharge (Butterworth et al. 2018). Although PONV is not a fatal complication, it can cause more significant disturbances than postoperative pain (Macario et al. 1999; Tramer 2003). To date, a significant amount of research has been dedicated to the identification of risk factors for PONV (Apfel et al. 1998; Apfel et al. 2002a; Apfel Christian et al. 1999; Apipan et al. 2016; Gan 2002; Gan 2006; Holder-Murray et al. 2019; Kim et al. 2020a; Kim et al. 2020b; Kim et al. 2020c; Kwon et al. 2020; Watcha and White 1992) and the establishment of guidelines for the management of PONV (Gan et al. 2019; Gan et al. 2014). However, there is no consensus regarding the risk factors for PONV.

The American Society of Anesthesiologists Physical Status (ASA-PS) Classification System is the most commonly used tool for the preoperative assessment of patients (Ahmed et al. 2019). This system was developed as a simple classification tool to evaluate a patient’s physiological condition and help predict surgical risk. Anesthesiologists use this scale to examine a patient’s health status and surgical risk (Doyle and Garmon 2019). Reports to date show that ASA-PS scores may be conflicting with respect to the occurrence of PONV (Gan et al. 2019; Gan et al. 2014). However, because the score is affected by some conditions that influence the choice of anesthetic agents, the pharmacokinetics of the medications used (Butterworth et al. 2018; St Pierre et al. 2000; Hines and Jones 2021; Freye and Levy 2004), postoperative management, and the development of complications, we hypothesized that the ASA-PS score can affect the development of PONV. Therefore, we analyzed the relationship between the ASA-PS score and PONV development using propensity score matching and a proportional hazard model in a large patient group.

## Methods

### Ethical approval/informed consent

This study was approved by the Clinical Research Ethics Committee of Chuncheon Sacred Heart Hospital, Hallym University (IRB No. 2021-01-012). The study included vulnerable participants, but because it was a retrospective analysis of clinical data acquired in the treatment process that had already been completed, the informed consent of all clinical trial subjects was exempted from the research approval of the institution.

### Data sources

All data were obtained from the clinical data warehouse (CDW) of five hospitals of the Hallym University Medical Center. The CDW is a database of medical records, prescriptions, and test results from Hallym University Medical Center, containing 6 years (January 1, 2015, to December 31, 2020) of outpatient and inpatient data. Patients can be searched based on prescriptions, examinations, and diagnosis, among other variables. The CDW can provide medical records in an unstructured format in addition to the patient’s test, transfusion, and drug administration records.

### Study design and setting

We conducted this retrospective, matched cohort study from January 2015 to May 2020. The ASA House of Delegates approved the latest version of the ASA-PS Classification System on October 15, 2014 (Doyle and Garmon 2019). The current study included APA-PS data from January 1, 2015, a month and a half after the release of the latest version of ASA-PS considering the period of introduction and conversion of ASA-PS in each hospital. This manuscript adheres to the applicable STROBE guidelines.

### Participants

We included patients who underwent procedures under anesthesia (except for local anesthesia) and who were eligible for matching. Patients under 18 years of age, patients who were unconscious after surgery, patients with preoperative nausea and vomiting, patients who underwent reoperation or ventilator therapy within 24 h after surgery, and patients who had outlier or missing medical records were excluded. Patients were included as match eligibility if they underwent multiple procedures yet did not undergo reoperation within 24 h and did not experience PONV prior to anesthesia.

### Primary outcomes and secondary outcomes

Primary outcomes included PONV and postoperative vomiting (PV). Patients with a history of nausea and vomiting as reported in medical records were determined as positive results. PONV was defined as nausea or vomiting occurring in any order within 24 h after surgery. The occurrence time of the first event was set as the occurrence time of PONV. PV was defined as vomiting occurring regardless of nausea within 24 h after surgery. The time to onset of vomiting was set as the time to onset of vomiting after surgery, regardless of whether or not there was nausea within 24 h after surgery. Secondary outcomes were intraoperative invasive monitoring and the use of vasopressors or inotropes during the operation or in the recovery room. To determine whether there was a difference between intraoperative monitoring and drug use due to differences in ASA-PS scores, monitoring via arterial catheterization, central venous pressure monitoring, indwelling urinary catheter, and intraoperative vasopressor or inotrope use were compared before and after propensity score matching.

### Exposure variables

We divided the ASA-PS Classification System into three categories: ASA-PS 1, ASA-PS 2, and ASA-PS 3 and higher. When one ASA-PS score was used as the control exposure, the succeeding scores were evaluated as the primary exposure. For example, if the control exposure was ASA-PS 1, the primary exposure studied before anesthesia was ASA-PS 2 or ASA-PS 3 and higher. If the control exposure was ASA-PS 2, the primary exposure studied was ASA-PS 3 and higher. To minimize bias due to the cause of determining ASA-PS, we implemented a matched cohort design using propensity score matching.

### Other variables

Other perioperative covariates were used to adjust for confounding and bias in the determination of ASA-PS class. The covariates included PONV risk factors with positive clinical evidence or factors that could influence the determination of ASA-PS scores. The female sex; smoking status; young age (< 50 years); obesity; menstruation; Levin tube; the type of anesthesia; volatile anesthetics; the use of nitrous oxide for more than 1 h, opioids, steroid, neostigmine, anticholinergics, and antiemetics; laparoscopic surgery; the duration of anesthesia and recovery room stay; and the type of surgery (abdomen, gynecology, ophthalmology, otorhinolaryngology, and head and neck) were included. Opioid use was subdivided into opioid use in the operating and recovery rooms and opioid use after discharge from the recovery room. After converting opioids (except remifentanil) to the equivalent dose of morphine, the value divided by body weight was used as a covariate. The use of remifentanil was included as a covariate.

### Statistical methods

Continuous data are presented as median and interquartile ranges due to skew, while categorical data are presented as frequencies and percentages. Binary primary outcomes are summarized using frequencies and percentages for each matched group. Unadjusted differences among patients with each ASA-PS score were assessed using logistic regression as the estimation algorithm and the nearest neighbor algorithm as the matching algorithm to account for the matching. Variables used for matching were assessed for confounding using absolute standardized differences. Adjusted hazard ratios (AHRs) with 95% confidence intervals (CIs) were reported for all matching models with Cox proportional hazard regression. Two adjusted hazard ratios were calculated, one including all variables and one including all variables and the propensity score. All statistical analyzes were performed using SPSS version 26.0 (IBM, USA), and hypothesis testing was two-sided (alpha = 0.05).

### Sensitivity analysis

ASA-PS 2 is defined as a patient with mild systemic disease. However, according to the 2014 ASA-PS Classification System, obesity, smoking, and drinking are also included in the ASA-PS 2. However, our data included patients with ASA-PS 1 who were obese and smoked or drank alcohol, although they did not have any other conditions. Therefore, we conducted a sensitivity analysis by considering obesity, smoking, and drinking patients with ASA-PS 1 as ASA-PS 2. After performing propensity score matching in the same way as the original data, the risk ratio was calculated. Additionally, because of the confounding of smoking as being both a protective factor against PONV and a criterion for advancing ASA-PS score, we investigated the hazard ratio of ASA-PS between 1 and 2 in non-smokers using sensitivity analysis.

## Results

**Fig. 1:**
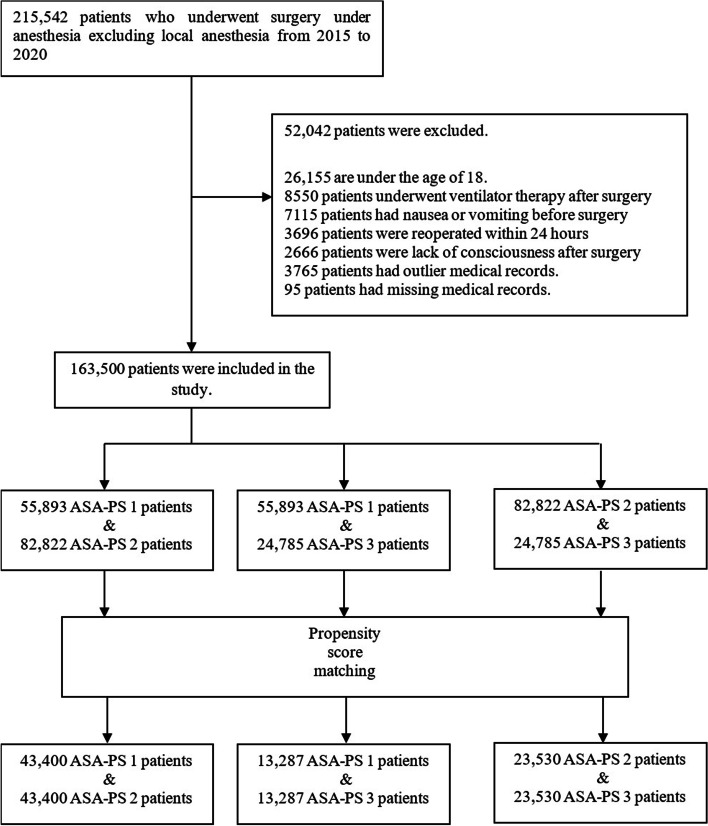
Flow chart of patient recruitment and matching. ASA, American Society of Anesthesiologists; PS, physical status

**Fig. 2:**
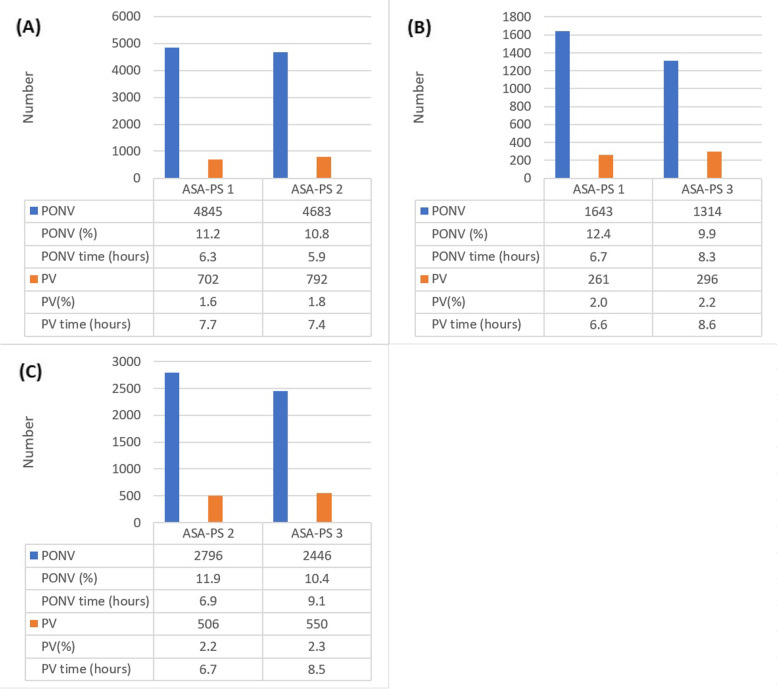
PONV and PV event number, rates, and event time. Postoperative nausea and vomiting and postoperative vomiting event number, rates, and event time in the matched cohort of patients who underwent procedures under anesthesia, excluding local anesthesia. **A** ASA-PS 1 vs. 2. **B** ASA-PS 1 vs. 3. **C** ASA-PS 2 vs. 3. PONV, postoperative nausea and vomiting; PV, postoperative vomiting; ASA, American Society of Anesthesiologists; PS, physical status

Among 215,542 eligible cases, 52,042 were excluded, and 163,500 patients’ complete outcomes and perioperative data were eligible for matching. The reasons for and the number of excluded patients and the number of patients in each ASA-PS class before and after matching are summarized in Fig. 1. Covariates before and after matching used in the proportional hazard model are summarized in Table 1 (ASA-PS 1 vs. 2), Table 2 (ASA-PS 1 vs. 3 and higher), and Table 3 (ASA-PS 2 vs. 3 and higher). Before matching, PONV occurred 5980, 9257, and 2591 in ASA-PS 1, ASA 2, and ASA-PS 3 and higher patients, respectively. PV occurred 863, 1600, and 584 in ASA-PS 1, ASA 2, and ASA-PS 3 and higher patients, respectively. The median times to PONV occurrence were 6.3 (IQR, 2.3–12.7), 6.2 (2.1–14.0), and 6.8 (2.3–15.6) h after surgery in ASA-PS 1, 2, and 3 and higher, respectively. The median times to PV occurrence were 7.7 (2.5–13.5), 7.9 (2.9–15.4), and 8.5 (3.7–16.8) h after surgery in ASA-PS 1, 2, and 3 and higher, respectively. PONV and PV event rates and event time in the matched cohort of patients are summarized in Fig. 2.

**Table 1:** Characteristics and perioperative data before and after propensity score matching of ASA-PS 1 and 2 patients

	Before matching			After matching		
	ASA PS 1 (*n* = 55,893)	ASA PS 2 (*n* = 82,822)	ASD	ASA PS 1 (*n* = 43,400)	ASA PS 2 (*n* = 43,400)	ASD
Young age (< 50)	41,342 (74.0)	32,061 (38.7)	0.72	28,849 (66.5)	27,588 (63.6)	0.06
Female	28,933 (51.8)	42,983 (51.9)	0	22,588 (52.0)	22,727 (52.4)	0.01
Obesity	2462 (4.4)	8913 (10.8)	0.21	2462 (5.7)	2728 (6.3)	0.02
Smoking	11,801 (21.1)	14,761 (17.8)	0.09	8899 (20.5)	8764 (20.2)	0.01
Menstruation	93 (0.2)	43 (0.1)	0.05	38 (0.1)	39 (0.1)	0
Levin tube	243 (0.4)	1499 (1.8)	0.1	243 (0.6)	330 (0.8)	0.02
General anesthesia	46,047 (82.4)	69,513 (83.9)	0.04	35,489 (81.8)	35,856 (82.6)	0.02
Inhalation anesthetics	42,782 (76.5)	64,464 (77.8)	0.03	32,768 (75.5)	33,234 (76.6)	0.03
N_2_O	5947 (10.6)	7500 (9.1)	0.06	4182 (9.6)	3984 (9.2)	0.02
Remifentanil	24,888 (44.5)	49,275 (59.5)	0.3	22,877 (52.7)	23,421 (54.0)	0.03
Steroid	2285 (4.1)	3647 (4.4)	0.02	1771 (4.1)	1768 (4.1)	0
Neostigmine	7546 (13.5)	17,197 (20.8)	0.18	7414 (17.1)	8037 (18.5)	0.04
Anticholinergics	45,809 (82.0)	68,716 (83.0)	0.03	35,234 (81.2)	35,563 (81.9)	0.02
Antiemetics	41,048 (73.4)	64,851 (78.3)	0.12	32,232 (74.3)	32,482 (74.8)	0.01
Laparoscopic surgery	14,841 (26.6)	17,392 (21.0)	0.14	10,288 (23.7)	10,286 (23.7)	0
Abdominal surgery	11,133 (19.9)	15,353 (18.5)	0.04	7965 (18.4)	8027 (18.5)	0
GY surgery	7756 (13.9)	8713 (10.5)	0.11	6083 (14.0)	6011 (13.9)	0.01
EYE surgery	438 (0.8)	1072 (1.3)	0.05	416 (1.0)	443 (1.0)	0.01
ENT surgery	7819 (14.0)	8139 (9.8)	0.14	5193 (12.0)	5150 (11.9)	0
Head and neck surgery	2553 (4.6)	4149 (5.0)	0.02	2185 (5.0)	2237 (5.2)	0.01
Anesthesia time (h)	1.4 (1.0, 2.2)	1.8 (1.1, 2.7)	0.25	1.5 (1.0, 2.3)	1.6 (1.1, 2.3)	0.03
Recovery room time (h)	0.5 (0.4, 0.6)	0.5 (0.5, 6.0)	0.13	0.5 (0.4, 0.6)	0.5 (0.4, 0.6)	0.02
NPO time (h)	11.5 (9.2, 14.0)	11.2 (8.8, 13.6)	0.11	11.3 (9.0, 13.8)	11.3 (9, 13.8)	0.01
Input and output (ml/kg)	5.2 (3.2, 8.2)	6.0 (3.5, 10.0)	0.19	5.5 (3.4, 8.7)	5.5 (3.3, 9.0)	0.02
Opioid in OR and RR (mg/kg)	5.0 (0.3, 7.5)	4.0 (0.2, 5.8)	0.09	4.0 (0.2, 6.3)	4.0 (0.0, 6)	0
Opioid after RR (mg/kg)	0.0 (0.0, 47.3)	0.0 (0.0, 94.3)	0.18	0.0 (0.0, 66.9)	0.0 (0.0, 73.5)	0.03

Values are number (percentages) or median (interquartile ranges)

*ASA* American Society of Anesthesiologists, *PS* physical status, *GY* gynecology, *ENT* otorhinolaryngology, *NPO* nothing by mouth, *OR* operation room, *RR* recovery room, *ASD* absolute standardized differences

**Table 2:** Characteristics and perioperative data before and after propensity score matching of ASA-PS 1 and 3 patients

	Before matching			After matching		
	ASA PS 1 (*n* = 55,893)	ASA PS 3 (*n* = 24,785)	ASD	ASA PS 1 (*n* = 13,287)	ASA PS 3 (*n* = 13,287)	ASD
Young age (< 50)	41,342 (74.0)	2866 (11.6)	1.95	2834 (21.3)	2770 (20.8)	0.02
Female	28,933 (51.8)	11,556 (46.6)	0.1	6450 (48.5)	6448 (48.5)	0
Obesity	2462 (4.4)	2048 (8.3)	0.14	781 (5.9)	861 (6.5)	0.02
Smoking	11,801 (21.1)	3358 (13.5)	0.22	2253 (17.0)	2153 (16.2)	0.02
Menstruation	93 (0.2)	5 (0.0)	0.1	7 (0.1)	5 (0.0)	0.01
Levin tube	243 (0.4)	929 (3.7)	0.17	165 (1.2)	218 (1.6)	0.02
General anesthesia	46,047 (82.4)	22,331 (90.1)	0.26	11,055 (83.2)	11,065 (83.3)	0
Inhalation anesthetics	42,782 (76.5)	20,346 (82.1)	0.14	9985 (75.1)	9995 (75.2)	0
N_2_O	5947 (10.6)	1518 (6.1)	0.19	1294 (9.7)	1162 (8.7)	0.04
Remifentanil	24,888 (44.5)	18,580 (75.0)	0.7	7766 (58.4)	8007 (60.3)	0.04
Steroid	2285 (4.1)	1204 (4.9)	0.04	545 (4.1)	557 (4.2)	0
Neostigmine	7546 (13.5)	9153 (36.9)	0.49	2743 (20.6)	2912 (21.9)	0.03
Anticholinergics	45,809 (82.0)	21,336 (86.1)	0.12	10,837 (81.6)	10,774 (81.1)	0.01
Antiemetics	41,048 (73.4)	21,357 (86.2)	0.37	10,834 (81.5)	10,868 (81.8)	0.01
Laparoscopic surgery	14,841 (26.6)	4524 (18.3)	0.21	2395 (18.0)	2421 (18.2)	0.01
Abdominal surgery	11,133 (19.9)	5306 (21.4)	0.04	2390 (18.0)	2512 (18.9)	0.02
GY surgery	7756 (13.9)	746 (3.0)	0.64	736 (5.5)	648 (4.9)	0.04
EYE surgery	438 (0.8)	275 (1.1)	0.03	178 (1.3)	167 (1.3)	0.01
ENT surgery	7819 (14.0)	1361 (5.5)	0.37	1278 (9.6)	1198 (9.0)	0.03
Head and neck surgery	2553 (4.6)	830 (3.3)	0.07	573 (4.3)	537 (4.0)	0.02
Anesthesia time (h)	1.4 (1.0, 2.2)	2.1 (1.3, 3.1)	0.47	1.8 (1.2, 2.7)	1.7 (1.1, 2.6)	0.03
Recovery room time (h)	0.5 (0.4, 0.6)	0.6 (0.4, 0.7)	0.01	0.6 (0.4, 0.7)	0.5 (0.4, 0.6)	0
NPO time (h)	11.5 (9.2, 14.0)	11.3 (8.8, 13.7)	0.09	11.4 (8.9, 13.8)	11.2 (8.9, 13.8)	0
Input and output (ml/kg)	5.2 (3.2, 8.2)	8.1 (4.4, 14.5)	0.42	6.3 (3.5, 10.8)	6.2 (3.8, 9.8)	0.04
Opioid in OR and RR (mg/kg)	5.0 (0.3, 7.5)	2.8 (0.0, 5.0)	0.4	2.9 (0.0, 5.3)	3.1 (0.0, 5.5)	0.03
Opioid after RR (mg/kg)	0.0 (0, 47.3)	36.1 (0.0, 175.8)	0.37	0.0 (0.0, 123.0)	0.0 (0.0, 97.0)	0.03

Values are number (percentages) or median (interquartile ranges)

*ASA* American Society of Anesthesiologists, *PS* physical status, *GY* gynecology, *ENT* otorhinolaryngology, *NPO* nothing by mouth, *OR* operation room, *RR* recovery room, *ASD* absolute standardized differences

**Table 3:** Characteristics and perioperative data before and after propensity score matching of ASA-PS 2 and 3 patients

	Before matching			After matching		
	ASA PS 2 (*n* = 82,822)	ASA PS 3 (*n* = 24,785)	ASD	ASA PS 2 (*n* = 23,530)	ASA PS 3 (*n* = 23,530)	ASD
Young age (< 50)	32,061 (38.7)	2866 (11.6)	0.85	3038 (12.9)	2862 (12.2)	0.02
Female	42,983 (51.9)	11,556 (46.6)	0.11	10,975 (46.6)	11,078 (47.1)	0.01
Obesity	8913 (10.8)	2048 (8.3)	0.09	2028 (8.6)	1976 (8.4)	0.01
Smoking	14,761 (17.8)	3358 (13.5)	0.12	3345 (14.2)	3264 (13.9)	0.01
Menstruation	43 (0.1)	5 (0.0)	0.02	6 (0.0)	5 (0.0)	0
Levin tube	1499 (1.8)	929 (3.7)	0.1	784 (3.3)	784 (3.3)	0
General anesthesia	69,513 (83.9)	22,331 (90.1)	0.21	21,088 (89.6)	21,078 (89.6)	0
Inhalation anesthetics	64,464 (77.8)	20,346 (82.1)	0.11	19,249 (81.8)	19,253 (81.8)	0
N_2_O	7500 (9.1)	1518 (6.1)	0.12	1498 (6.4)	1501 (6.4)	0
Remifentanil	49,275 (59.5)	18,580 (75.0)	0.36	17,401 (74.0)	17,349 (73.7)	0.01
Steroid	3647 (4.4)	1204 (4.9)	0.02	1090 (4.6)	1082 (4.6)	0
Neostigmine	17,197 (20.8)	9153 (36.9)	0.33	8146 (34.6)	8159 (34.7)	0
Anticholinergics	68,716 (83.0)	21,336 (86.1)	0.09	20,284 (86.2)	20,186 (85.8)	0.01
Antiemetics	64,851 (78.3)	21,357 (86.2)	0.23	20,213 (85.9)	20,140 (85.6)	0.01
Laparoscopic surgery	17,392 (21.0)	4524 (18.3)	0.07	4374 (18.6)	4350 (18.5)	0
Abdominal surgery	15,353 (18.5)	5306 (21.4)	0.07	4868 (20.7)	4934 (21.0)	0.01
GY surgery	8713 (10.5)	746 (3.0)	0.44	794 (3.4)	743 (3.2)	0.01
EYE surgery	1072 (1.3)	275 (1.1)	0.02	262 (1.1)	274 (1.2)	0
ENT surgery	8139 (9.8)	1361 (5.5)	0.19	1368 (5.8)	1361 (5.8)	0
Head and neck surgery	4149 (5.0)	830 (3.3)	0.09	816 (3.5)	825 (3.5)	0
Anesthesia time (h)	1.8 (1.1, 2.7)	2.1 (1.3, 3.1)	0.25	1.9 (1.3, 3.1)	2.0 (1.3, 3.0)	0
Recovery room time (h)	0.5 (0.5, 6.0)	0.6 (0.4, 0.7)	0.09	0.6 (0.5, 0.6)	0.6 (0.4, 0.7)	0.01
NPO time (h)	11.2 (8.8, 13.6)	11.3 (8.8, 13.7)	0.01	11.2 (8.8, 13.7)	11.2 (8.8, 13.7)	0
Input and output (ml/kg)	6.0 (3.5, 10.0)	8.1 (4.4, 14.5)	0.28	7.3 (4.3, 12.4)	7.7 (4.3, 13.6)	0.01
Opioid in OR and RR (mg/kg)	4.0 (0.2, 5.8)	2.8 (0.0, 5.0)	0.29	3.1 (0.0, 5.0)	2.8 (0.0, 5.0)	0.03
Opioid after RR (mg/kg)	0.0 (0.0, 94.3)	36.1 (0.0, 175.8)	0.22	0.0 (0.0, 165.0)	32.1 (0.0, 166.7)	0.01

Values are number (percentages) or median (interquartile ranges)

*ASA* American Society of Anesthesiologists, *PS* physical status, *GY* gynecology, *ENT* otorhinolaryngology, *NPO* nothing by mouth, *OR* operation room, *RR* recovery room, *ASD* absolute standardized differences

In the sensitivity analysis, ASA-PS 1, 2, and 3 and higher patients were 31,598, 107,117, and 24,278, respectively. The matchings of each ASA-PS were performed to 1:1. After matching of ASA-PS 1 vs. 2, 1 vs. 3 and higher, and 2 vs. 3 and higher, patients were 31,091, 8773, and 23,614, respectively. Before matching, PONV occurred 4266, 10,971, and 2591 in ASA-PS 1, ASA 2, and ASA-PS 3 and higher patients, respectively. PV occurred 656, 1807, and 584 in ASA-PS 1, ASA 2, and ASA-PS 3 and higher patients, respectively. The median times to PONV occurrence before matching were 6.3 (IQR, 2.3–12.3), 6.2 (2.1–14.0), and 6.8 (2.3–15.6) h after surgery in ASA-PS 1, 2, and 3 and higher, respectively. The median times to PV occurrence before matching were 7.7 (2.7–13.2), 7.9 (2.7–15.4), and 8.5 (3.7–16.8) h after surgery in ASA-PS 1, 2, and 3 and higher, respectively. The absolute standardized differences of covariates before matching were 0.00 to 0.57, 0.02 to 1.84, and 0.00 to 1.13 in ASA-PS 1 vs. 2, 1 vs. 3 and higher, and 2 vs. 3 and higher, respectively. After matching, the absolute standardized differences were lower than 0.1 in all matching. The details are summarized in online Additional files 1, 2 and 3.

ASA-PS 3 and higher was associated with a 24% (AHR, 0.76; 95% CI, 0.71 to 0.82) and 14% (AHR, 0.86; 95% CI, 0.82 to 0.91) reduced risk of PONV than ASA-PS 1 and 2, respectively (Table 4). ASA-PS 2 was associated with a 12% (AHR, 1.12; 95% CI, 1.01 to 1.25) increased risk of PV than ASA-PS 1. Sensitivity analyses demonstrated similar results in ASA 1 vs. 3 and higher, and 2 vs. 3 and higher, but showed different results in ASA 1 vs. 2. ASA-PS 2 was associated with an 8% (AHR, 0.92; 95% CI, 0.88 to 0.96) reduced risk of PONV than ASA-PS 1. The ratio of invasive arterial pressure monitoring, central venous pressure monitoring, and indwelling urinary catheterization was higher in high ASA-PS classification than in low ASA-PS classification regardless of matching. The number and ratio of invasive arterial pressure monitoring, central venous pressure monitoring, and indwelling urinary catheterization are summarized in Table 5 (before matching) and Table 6 (after matching). Sensitivity analysis also showed that the ratios were higher in the high ASA-PS class than in the low ASA-PS class, regardless of the matching. In sensitivity analysis of non-smoker status between ASA-PS 1 and ASA-PS 2, ASA-PS 2 had an increased risk of PONV (UHR [95% CI], 0.93 [0.89 to 0.97]; AHR with all variables [95% CI], 0.93 [0.89 to 0.97]) than ASA-PS 1, but there was no increase in PV (UHR [95% CI], 1.05 [0.93 to 1.16]; AHR with all variables [95% CI], 1.04 [0.93 to 1.16]).

**Table 4:** Unadjusted, all variables adjusted, and all variables and propensity score-adjusted Association of American Society of Anesthesiologists Physical Status (ASA-PS)

			HR (95% CI) ASA PS 1 vs. 2	*P* value	HR (95% CI) ASA PS 1 vs. 3	*P* value	HR (95% CI) ASA PS 2 vs. 3	*P* value
Original data analysis	PONV	Unadjusted	0.97 (0.93–1.01)	0.09	0.79 (0.74–0.85)	< 0.001	0.87 (0.82–0.92)	< 0.001
		AV adjusted	0.96 (0.93–1.0)	0.07	0.76 (0.71–0.82)	< 0.001	0.86 (0.82–0.91)	< 0.001
		AVP adjusted	0.96 (0.93–1.0)	0.07	0.76 (0.71–0.82)	< 0.001	0.86 (0.82–0.91)	< 0.001
	PV	Unadjusted	1.13 (1.02–1.25)	0.02	1.14 (0.96–1.34)	0.14	1.09 (0.96–1.23)	0.17
		AV adjusted	1.12 (1.02–1.25)	0.02	1.08 (0.92–1.28)	0.35	1.07 (0.95–1.21)	0.26
		AVP adjusted	1.12 (1.01–1.25)	0.02	1.08 (0.92–1.28)	0.34	1.08 (0.95–1.21)	0.24
Sensitivity analysis	PONV	Unadjusted	0.92 (0.88–0.96)	< 0.001	0.69 (0.63–0.75)	< 0.001	0.86 (0.81–0.91)	< 0.001
		AV adjusted	0.92 (0.88–0.96)	< 0.001	0.68 (0.63–0.74)	< 0.001	0.85 (0.81–0.9)	< 0.001
		AVP adjusted	0.92 (0.88–0.96)	< 0.001	0.68 (0.63–0.74)	< 0.001	0.86 (0.81–0.9)	< 0.001
	PV	Unadjusted	1.02 (0.92–1.14)	0.72	0.99 (0.82–1.19)	0.88	1.1 (0.97–1.24)	0.13
		AV adjusted	1.02 (0.91–1.14)	0.74	0.96 (0.80–1.16)	0.7	1.08 (0.96–1.22)	0.19
		AVP adjusted	1.02 (0.92–1.14)	0.7	0.96 (0.80–1.16)	0.69	1.09 (0.96–1.23)	0.17

In a matched cohort of patients, the association of ASA-PS class with PONV and PV was assessed using multivariable Cox proportional hazard regression

*PONV* postoperative nausea and vomiting, *PV* postoperative vomiting, *HR* hazard ratio, *CI* confidence interval, *ASA-PS* American Society of Anesthesiologists Physical Status, *AV* all variables, *AVP* all variables + propensity score

**Table 5:** Number and percentages of monitoring and use of vasopressors or inotropes before propensity score matching

		ASA-PS 1	ASA-PS 2	ASA-PS 3	ASD (ASA-PS 1 vs. 2/1 vs. 3/2 vs. 3)
Original data analysis	IAM	5292 (9.5)	21,406 (25.8)	17,693 (71.4)	0.73/1.90/1.55
	CVP	624 (1.1)	4748 (5.7)	4381 (17.7)	0.98/0.36/0.77
	IUC	12,989 (23.2)	30,053 (36.3)	13,732 (55.4)	0.39/0.86/0.46
	VI	16,563 (29.6)	36,423 ( 44.0)	17,080 (68.9)	0.39/1.00/0.60
Sensitivity analysis	IAM	3163 (10.0)	23,488 (22.0)	17,740 (70.9)	0.52/1.86/1.23
	CVP	381 (1.2)	4981 (4.7)	4390 (17.5)	0.74/1.67/0.88
	IUC	8708 (27.6)	34,246 (32.0)	13,820 (55.2)	0.12/0.73/0.55
	VI	9823 (31.1)	43,076 (40.3)	17,168 (68.9)	0.23/0.98/0.66

*ASA* American Society of Anesthesiologists, *PS* physical status, *IAM* invasive arterial monitoring, *CVP* central venous pressure monitoring, *IUC* indwelling urinary catheterization, *VI* use of vasopressors or inotropes, *ASD* absolute standardized difference

**Table 6:** Number and percentages of monitoring and use of vasopressors or inotropes after propensity score matching

		Original data analysis			Sensitivity analysis		
		Low ASA-PS	High ASA-PS	ASD	Low ASA-PS	High ASA-PS	ASD
ASA-PS 1 vs. 2	IAM	4885 (11.3)	8391 (19.3)	0.4	3148 (10.1)	5226 (16.9)	0.36
	CVP	600 (1.4)	1456 (3.4)	0.56	381 (1.2)	876 (2.8)	0.53
	IUC	10,629 (24.5)	14,668 (33.8)	0.28	8506 (27.4)	10,561 (34.0)	0.20
	VI	13,443 (31.0)	16,388 (37.8)	0.19	9679 (31.2)	11,487 (36.9)	0.16
ASA-PS 1 vs. 3	IAM	2205 (17.0)	8101 (61.0)	1.25	1421 (16.2)	5339 (60.9)	1.28
	CVP	393 (3.0)	1529 (11.5)	0.88	250 (2.8)	949 (10.8)	0.87
	IUC	9176 (33.0)	12,689 (53.9)	0.67	2183 (24.9)	4256 (48.5)	0.65
	VI	4970 (37.4)	9837 (59.0)	0.55	3373 (38.4)	5217 (59.5)	0.53
ASA-PS 2 vs. 3	IAM	8612 (36.6)	16,497 (70.1)	0.87	8627 (36.1)	16,667 (69.7)	0.87
	CVP	2324 (5.9)	3854 (16.4)	0.36	2301 (9.6)	3895 (16.3)	0.38
	IUC	9176 (39.0)	12,689 (53.9)	0.38	9242 (38.7)	12,872 (53.9)	0.39
	VI	12,951 (55.0)	15,934 (67.7)	0.34	12,937 (54.1)	16,128 (67.5)	0.35

*ASA* American Society of Anesthesiologists, *PS* physical status, *IAM* invasive arterial monitoring, *CVP* central venous pressure monitoring, *IUC* indwelling urinary catheterization, *VI* use of vasopressors or inotropes, *ASD* absolute standardized difference

## Discussion

In this study, we utilized a retrospective, matched cohort analysis to determine the relationship between preoperative medical status, assessed by ASA-PS, and the risk of PONV and PV development. Patients with ASA-PS 3 and higher showed a lower risk of PONV development than those with ASA-PS 1 and 2 (24% vs. 14%, respectively). However, there was no significant difference in the risk of PV. Our findings suggest that the patient’s physiological condition prior to surgery affected postoperative outcomes, particularly PONV occurrence. The matching algorithm resulted in a good balance across the studied groups in terms of patients, anesthesia, procedures, and medications (Tables 1, 2, and 3 [original data] and Supplementary files 1, 2 and 3 [sensitivity analysis]). Using risk factors with positive clinical evidence as covariates, we performed sensitivity analysis after considering that non-disease patients with obesity, smoking, and drinking may be included in ASA-PS 2 or 3. In sensitivity analysis, patients with ASA-PS 3 and higher showed a 32% and 14% lower risk of PONV development than did those with ASA-PS 1 and 2, respectively, and patients with ASA-PS 3 and higher did not have a significant difference in PV risk with did those with ASA-PS 1 and 2. The overall results revealed that the PONV risk in patients with ASA-PS 3 and higher was lower than that in patients with ASA-PS 1 and 2, while the risk of PV was not significantly different. The high ASA-PS classification used more intraoperative monitoring and vasopressor or inotropes than the low ASA-PS classification before and after matching. The results showed that, controlling similar perioperative factors at high ASA-PS scores, there may be many patients with conditions that require more intraoperative monitoring and the use of vasopressors or inotropes.

Previous studies included a better general condition as a risk factor of PONV (Kim et al. 2020a; Kwon et al. 2020; Cohen et al. 1994; Koivuranta et al. 1997). However, in one study, researchers examined PONV 72 h after surgery but failed to consider event occurrence time and postoperative factors despite the long observation time (Cohen et al. 1994). In another study, the PONV incidence pattern according to time was similar to our study, but no statistical analysis was performed (Koivuranta et al. 1997). Furthermore, previous researchers have not investigated PONV with the ASA-PS score as the primary risk factor. Comparisons were made between ASA-PS 1 and other scores or between ASA-PS 1–2 and ASA-PS 3 or higher; however, no comparisons were made between ASA-PS 2 or 3 and higher ASA-PS scores.

We found that patients with ASA-PS 3 and higher had a lower risk of developing PONV than ASA-PS 1 to 2, which was consistent with the findings from previous studies (Cohen et al. 1994; Koivuranta et al. 1997). However, according to whether non-disease patients with obesity, smoking, and drinking are included in ASA-PS 2 or 3, there were statistical differences in the association between ASA-PS classification. In this study, the ASA-PS classification used in sensitivity analysis may be more suitable for a recent version. In sensitivity analysis, a high ASA-PS classification score had a lower risk of PONV than a low ASA-PS classification score. These results showed consistency of the association between ASA-PS classification and PONV.

Vomiting is more fatal than nausea. When PV was analyzed separately, most results showed that PV was not associated with ASA-PS class. There were different results only between ASA-PS 1 and 2. However, when sensitivity analysis was performed, the results showed consistency of no association between ASA-PS class and PV. In particular, because smoking and obesity are factors known to reduce PONV risk (Sinclair et al. 1999; Sweeney 2002), they may have affected the differences. The differences in the association between ASA-PS classification and PONV and between ASA-PS classification and PV may be due to the effect of ASA-PV on postoperative nausea. Nausea is more common than vomiting. Considering the difference between nausea and vomiting, there may be a difference in the association with ASA-PS (Apfel et al. 2002b; Knapp and Beecher 1956). Nausea is a subjective sensation that must be assessed by the patient, not the observer. Nausea is best described as a desire to vomit without expelling muscle movement. As a subjective sensation, nausea should be considered a conscious cortical activity that may not affect the brainstem. Vomiting is a brainstem reflex and is not necessarily an exacerbated form of nausea, as nausea can occur without vomiting. Therefore, vomiting should be assessed independently. However, since nausea is difficult to distinguish and is frequently correlated with vomiting, vomiting without nausea is rare and may be a potential emetic phenomenon. In our study, nausea was evaluated as a result of inclusion in PONV (Gan 2006; Apfel et al. 2002b; Knapp and Beecher 1956).

Some preoperative conditions of patients that affect ASA-PS influence the choice of anesthetic agents, the pharmacokinetics of the medications used, the postoperative management, and the development of complications. In patients with increased intracranial pressure, intravenous anesthetic agents, such as propofol, are preferred to inhalation anesthetic agents (Butterworth et al. 2018). Etomidate is used in patients with hemodynamic instability. In short surgeries, etomidate increases PV (St Pierre et al. 2000). Sevoflurane may be associated with nephrotoxicity related to its fluoride content, although there is no definitive evidence. Thus, some anesthesiologists may not prefer sevoflurane in patients with kidney disease (Butterworth et al. 2018). In obese patients, highly lipophilic drugs, such as fentanyl and sufentanil, accumulate in fatty tissues when they are administered via infusion over long periods. Usually, highly lipophilic drugs show a significant increased volume of distribution in obese patients, and it seems that the dosing of these medications should be based on total body weight. However, because the majority of these drugs can accumulate in adipose tissues over time, prolonged effects can be seen (Hines and Jones 2021). Reduction in stroke volume can lead to protracted redistribution of opioids to the liver. This results in prolonged metabolization and lesser inactivation over time, followed by an increase in the duration of effects (Freye and Levy 2004).

We used risk factors with positive clinical evidence as covariates and found that most of them were related to anesthesia and surgery. Inhalation anesthetics, including N_2_O, are well-known risk factors of PONV. Anesthesiologists may attempt to use the least amount of anesthetics as possible for maintaining blood pressure in patients by using vasopressor or inotrope. Because higher ASA-PS classification used more vasopressor or inotrope, less anesthetics may be used in patients with higher ASA-PS classification. Patients with reduced levels of consciousness due to trauma or cerebrovascular injury, or patients with neurodegenerative changes due to dementia or other cognitive conditions, have reduced anesthesia requirements (Aranake et al. 2013). Hypoxia, acute metabolic acidosis, and acute hemorrhagic hypotension all cause a reduction of approximately 10 to 50% in the initial monitored anesthesia care (MAC) (Eger et al. 1965). However, drugs used for anesthesia and postsurgical recovery have a short duration of action or a short context-sensitive half time (Butterworth et al. 2018; Bailey 1997; Egan 1995). In this study, over 75% of PONV occurred 2 h post-anesthetic and over 50% occurred 5 h post-anesthetic. Therefore, the effects may be limited and not evident from surgery to the recovery room. In addition, our findings suggest that other risk factors not included in this study should be considered for PONV developing. More than three-quarters of PONV occurred 2 h post-anesthetic. In cases of PONV occurring 2 h post-anesthetic, antibiotic, and nonsteroidal anti-inflammatory drug use, the severity of postoperative pain, the postoperative fasting period, and the oxygen supply must be considered. Patients with high ASA-PS scores who have asthma or renal disease may have difficulty with nonsteroidal anti-inflammatory drugs, those with cardiopulmonary disease may require prolonged oxygen therapy, and those with aspiration risk may require prolonged fasting periods. The postsurgical role of these factors remains controversial (Gan 2006; Gan et al. 2019; Gan et al. 2014; Junger et al. 2001; Kearney et al. 1998; Stadler et al. 2003; Tramer 2001), but the association between ASA-PS class and these factors also needs to be considered.

The strength of this study was that a large number of patients were analyzed. Considering that there may be a difference between vomiting and nausea, the analysis of PV was separated. However, this study has several limitations. First, the reliability of the ASA-PS Classification System should be considered. ASA-PS is the most commonly used tool to classify the preoperative condition. It was created in 1941, revised in 1961, and revised again in 2014 (Mayhew et al. 2019). Although definitions and examples are described for ASA-PS, there are bound to be limitations in classifying all patients by these definitions and examples, and differences may arise due to these limitations. Therefore, it is difficult to maintain consistency in classification; this has already been reported in several studies (Haynes and Lawler 1995; Owens et al. 1978). We included patients from 2015 by considering the 2014 revision. In our study, 15% of the total subjects should have belonged to ASA-PS class 2 or 3 because of smoking, obesity (ASA-PS 2, 30 ≤ BMI < 40; ASA-PS 3, 40 ≤ BMI), and social alcohol consumption; however, they belonged to ASA-PS 1. We performed a sensitivity analysis for reducing this uncertainty. In addition, we found that there may be a difference in the number of vulnerable patients according to ASA-PS classification through secondary outcomes (including intraoperative invasive monitoring and use of vasopressor or inotrope). A second limitation was in the method of data collection. Patients complain of PONV much more often than indicated in the chart (Cohen et al. 1994). Direct, specific questions report actual PONV much more than spontaneous patient reports (Apfel et al. 1998; Apfel et al. 2002c), and higher workloads of nurses or physicians may result in fewer vomiting events being reported (Sinclair et al. 1999). The nature and severity of PONVs collected in studies may affect the accuracy or applicability of independent risk factor detection (Gan 2006). Third, despite the importance of the effects of additional antiemetics on PONV, we used antiemetics as a binary variable. It is well known that additional antiemetics are effective against PONV. However, because our data concerning the history of PONV and details about antiemetics, such as the timing and number of administration, was insufficient, we could not analyze the effect of the additional use of antiemetics according to PONV risk. Additional antiemetics could affect the results, and it seems necessary to analyze the effects of the controlled use of additional antiemetics in a further study.

In conclusion, in an analysis that controlled for PONV-related anesthesia and surgical factors, ASA-PS 3 and higher was found to reduce the risk of PONV but was not related to PV. Despite the limitations, the findings of this study may help anesthesiologists determine the risk of PONV in patients undergoing surgery under anesthesia. Considering the times of PONV occurrence that we observed which suggest that the effects of anesthesia and surgery factors may be limited, the effects of postoperative factors on PONV may need to be considered. Future studies focusing on the association of PONV with preoperative physical status will be needed.

## Supplementary Information

**Additional file 1: Supporting Information Table 1.** Characteristics and perioperative data before and after propensity score matching of ASA-PS 1 and 2 patients who underwent procedures under anesthesia except for local anesthesia in the sensitivity analysis. Values are number (percentages) or median (interquartile ranges). ASA, American Society of Anesthesiologists; PS, physical status; GY, gynecology; ENT; otorhinolaryngology; NPO, nothing by mouth; OR, operation room; RR, recovery room; ASD, absolute standardised difference.

**Additional file 2: Supporting Information Table 2.** Characteristics and perioperative data before and after propensity score matching of ASA-PS 1 and 3 patients who underwent procedures under anesthesia except for local anesthesia in the sensitivity analysis. Values are number (percentages) or median (interquartile ranges). ASA, American Society of Anesthesiologists; PS, physical status; GY, gynecology; ENT; otorhinolaryngology; NPO, nothing by mouth; OR, operation room; RR, recovery room; ASD, absolute standardised difference.

**Additional file 3: Supporting Information Table 3.** Characteristics and perioperative data before and after propensity score matching of ASA-PS 2 and 3 patients who underwent procedures under anesthesia except for local anesthesia in the sensitivity analysis. Values are number (percentages) or median (interquartile ranges). ASA, American Society of Anesthesiologists; PS, physical status; GY, gynecology; ENT; otorhinolaryngology; NPO, nothing by mouth; OR, operation room; RR, recovery room; ASD, absolute standardised difference.

## Data Availability

The datasets used and/or analyzed during the current study are available from the corresponding author on reasonable request.

## Abbreviations

AHR: Adjusted hazard ratio
CDW: Clinical data warehouse
CI: Confidence interval
NRF: National Research Foundation
PONV: Postoperative nausea and vomiting
PV: Postoperative vomiting
