# Supplementary material for: Effect of the preoperative physical status on postoperative nausea and vomiting risk: a matched cohort study
Source: Perioper Med (Lond). 2022 Sep 6;11:31. doi: 10.1186/s13741-022-00264-1 (PMC9446728; doi:10.1186/s13741-022-00264-1)
Supplement: Supplementary file 1 — Additional file 1: Supporting Information Table 1. Characteristics and perioperative data before and after propensity score matching of ASA-PS 1 and 2 patients who underwent procedures under anesthesia except for local anesthesia in the sensitivity analysis. Values are number (percentages) or median (interquartile ranges). ASA, American Society of Anesthesiologists; PS, physical status; GY, gynecology; ENT; otorhinolaryngology; NPO, nothing by mouth; OR, operation room; RR, recovery room; ASD, absolute standardised difference. [file 13741_2022_264_MOESM1_ESM.docx]

**Supporting Information Table 1** Characteristics and perioperative data before and after propensity score matching of ASA-PS 1 and 2 patients who underwent procedures under anesthesia except for local anesthesia. Values are number (percentages) or median (interquartile ranges). ASA, American Society of Anesthesiologists; PS, physical status; GY, gynecology; ENT; otorhinolaryngology; NPO, nothing by mouth; OR, operation room; RR, recovery room; ASD, absolute standardised difference

|  | Before matching | | | After matching | | |
| --- | --- | --- | --- | --- | --- | --- |
|  | ASA PS 1  (n=31598) | ASA PS 2  (n=107117) | ASD | ASA PS 1  (n=31091) | ASA PS 2  (n=31091) | ASD |
| Young age (<50) | 22271 (70.5) | 50941 (47.7) | 0.46 | 21764 (70.0) | 20614 (66.3) | 0.07 |
| Female | 21934 (69.4) | 49833 (46.6) | 0.46 | 21428 (68.9) | 20665 (66.5) | 0.05 |
| Obesity | 0 (0.0) | 11145 (10.4) | 0.34 | 0 (0.0) | 0 (0.0) | <0.01 |
| Smoking | 0 (0.0) | 26510 (24.8) | 0.57 | 0 (0.0) | 0 (0.0) | <0.01 |
| Menstruation | 68 (0.2) | 67 (0.1) | 0.06 | 55 (0.2) | 43 (0.1) | 0.01 |
| Levin tube | 135 (0.4) | 1606 (1.5) | 0.09 | 135 (0.4) | 218 (0.7) | 0.03 |
| General Anesthesia | 26792 (84.8) | 88576 (82.9) | 0.05 | 26285 (84.5) | 25884 (83.3) | 0.03 |
| Inhalation Anesthetics | 24760 (78.4) | 82302 (77.0) | 0.03 | 24267 (78.1) | 23923 (76.9) | 0.03 |
| N2O | 3095 (9.8) | 10331 (9.7) | <0.01 | 3044 (9.8) | 2889 (9.3) | 0.02 |
| Remifentanil | 14961 (47.3) | 59094 (55.3) | 0.16 | 14946 (48.1) | 15520 (49.9) | 0.04 |
| Steroid | 1275 (4.0) | 4635 (4.3) | 0.01 | 1248 (4.0) | 1202 (3.9) | 0.01 |
| Neostigmine | 4699 (14.9) | 20024 (18.7) | 0.10 | 4698 (15.1) | 5184 (16.7) | 0.04 |
| Anticholinergics | 26569 (84.1) | 87778 (82.1) | 0.05 | 26065 (83.8) | 25702 (82.7) | 0.03 |
| Antiemetics | 23352 (73.9) | 82383 (77.1) | 0.07 | 22931 (73.8) | 22992 (74.0) | <0.01 |
| Laparoscopic surgery | 9462 (29.9) | 22724 (21.3) | 0.21 | 9143 (29.4) | 8468 (27.2) | 0.05 |
| Abdominal surgery | 6222 (19.7) | 20223 (18.9) | 0.02 | 6139 (19.7) | 6009 (19.3) | 0.01 |
| GY surgery | 6184 (19.6) | 10248 (9.6) | 0.34 | 5980 (19.2) | 5510 (17.7) | 0.05 |
| EYE surgery | 232 (0.7) | 1276 (1.2) | 0.04 | 232 (0.7) | 261 (0.8) | <0.01 |
| ENT surgery | 4273 (13.5) | 11653 (10.9) | 0.08 | 4089 (13.2) | 3867 (12.4) | 0.03 |
| Head & neck surgery | 1374 (4.3) | 5322 (5.0) | 0.03 | 1373 (4.4) | 1439 (4.6) | 0.01 |
| Anesthesia time (hour) | 85 (60.0, 135) | 100.0 (65.0, 150) | 0.20 | 85.0 (60, 135) | 90.0 (60, 140) | 0.04 |
| Recovery room time (hour) | 30 (25.0, 35) | 31.0 (25.0, 36) | 0.06 | 30.0 (25, 35) | 30.0 (25, 35) | 0.01 |
| NPO time (hour) | 11.4 (9.0, 13.9) | 11.2 (8.9, 13.8) | 0.05 | 11.3 (9, 13.9) | 11.3 (9, 13.8) | 0.01 |
| Input & output (ml/kg) | 5.5 (3.3, 8.6) | 5.7 (3.4, 9.4) | 0.10 | 5.5 (3.3, 8.7) | 5.6 (3.3, 9.0) | 0.03 |
| Opioid in OR & RR (mg/kg) | 4.6 (0.4, 7.5) | 4.0 (0.2, 6) | 0.05 | 4.6 (0.4, 7.5) | 4.0 (0.2, 6.4) | 0.03 |
| Opioid after RR (mg/kg) | 0 (0.0, 43.3) | 0.0 (0.0, 84.7) | 0.17 | 0.0 (0, 44.1) | 0.0 (0, 59.0) | 0.03 |
