# Supplementary material for: Effect of the preoperative physical status on postoperative nausea and vomiting risk: a matched cohort study
Source: Perioper Med (Lond). 2022 Sep 6;11:31. doi: 10.1186/s13741-022-00264-1 (PMC9446728; doi:10.1186/s13741-022-00264-1)
Supplement: Supplementary file 2 — Additional file 2: Supporting Information Table 2. Characteristics and perioperative data before and after propensity score matching of ASA-PS 1 and 3 patients who underwent procedures under anesthesia except for local anesthesia in the sensitivity analysis. Values are number (percentages) or median (interquartile ranges). ASA, American Society of Anesthesiologists; PS, physical status; GY, gynecology; ENT; otorhinolaryngology; NPO, nothing by mouth; OR, operation room; RR, recovery room; ASD, absolute standardised difference. [file 13741_2022_264_MOESM2_ESM.docx]

**Supporting Information Table 2** Characteristics and perioperative data before and after propensity score matching of ASA-PS 1 and 3 patients who underwent procedures under anesthesia except for local anesthesia. Values are number (percentages) or median (interquartile ranges). ASA, American Society of Anesthesiologists; PS, physical status; GY, gynecology; ENT; otorhinolaryngology; NPO, nothing by mouth; OR, operation room; RR, recovery room; ASD, absolute standardised difference

|  | Before matching | | | After matching | | |
| --- | --- | --- | --- | --- | --- | --- |
|  | ASA PS 1  (n=31598) | ASA PS 3  (n=25015) | ASD | ASA PS 1  (n=8773) | ASA PS 3  (n=8773) | ASD |
| Young age (<50) | 22271 (70.5) | 3057 (12.2) | 1.84 | 1647 (18.8) | 1554 (17.7) | 0.02 |
| Female | 21934 (69.4) | 11705 (46.8) | 0.46 | 5525 (63.0) | 5337 (60.8) | 0.06 |
| Obesity | 0 (0.0) | 2278 (9.1) | 0.30 | 0 (0.0) | 50 (0.6) | 0.02 |
| Smoking | 0 (0.0) | 3410 (13.6) | 0.40 | 0 (0.0) | 50 (0.6) | 0.02 |
| Menstruation | 68 (0.2) | 6 (0.0) | 0.14 | 3 (0.0) | 2 (0.0) | 0.03 |
| Levin tube | 135 (0.4) | 930 (3.7) | 0.17 | 96 (1.1) | 110 (1.3) | 0.01 |
| General Anesthesia | 26792 (84.8) | 22523 (90.0) | 0.18 | 7212 (82.2) | 7250 (82.6) | 0.01 |
| Inhalation Anesthetics | 24760 (78.4) | 20530 (82.1) | 0.10 | 6479 (73.9) | 6523 (74.4) | <0.01 |
| N2O | 3095 (9.8) | 1539 (6.2) | 0.15 | 775 (8.8) | 774 (8.8) | 0.01 |
| Remifentanil | 14961 (47.3) | 18688 (74.7) | 0.64 | 5042 (57.5) | 5131 (58.5) | 0.02 |
| Steroid | 1275 (4.0) | 1226 (4.9) | 0.04 | 343 (3.9) | 350 (4.0) | <0.01 |
| Neostigmine | 4699 (14.9) | 9173 (36.7) | 0.46 | 1818 (20.7) | 1941 (22.1) | 0.02 |
| Anticholinergics | 26569 (84.1) | 21514 (86.0) | 0.06 | 7084 (80.7) | 7119 (81.1) | 0.01 |
| Antiemetics | 23352 (73.9) | 21521 (86.0) | 0.35 | 7180 (81.8) | 7175 (81.8) | 0.01 |
| Laparoscopic surgery | 9462 (29.9) | 4571 (18.3) | 0.30 | 1686 (19.2) | 1652 (18.8) | 0.01 |
| Abdominal surgery | 6222 (19.7) | 5347 (21.4) | 0.04 | 1594 (18.2) | 1651 (18.8) | 0.02 |
| GY surgery | 6184 (19.6) | 783 (3.1) | 0.97 | 628 (7.2) | 529 (6.0) | 0.06 |
| EYE surgery | 232 (0.7) | 277 (1.1) | 0.04 | 109 (1.2) | 105 (1.2) | <0.01 |
| ENT surgery | 4273 (13.5) | 1393 (5.6) | 0.35 | 850 (9.7) | 813 (9.3) | 0.03 |
| Head & neck surgery | 1374 (4.3) | 836 (3.3) | 0.06 | 376 (4.3) | 383 (4.4) | 0.01 |
| Anesthesia time (hour) | 85 (60.0, 135) | 125.0 (80.0, 185.0) | 0.47 | 100.0 (65, 155) | 110.0 (70, 155) | 0.03 |
| Recovery room time (hour) | 30 (25.0, 35) | 35.0 (24.0, 39.0) | 0.02 | 30.0 (25, 35) | 33.0 (24, 39) | 0.01 |
| NPO time (hour) | 11.4 (9.0, 13.9) | 11.3 (8.8, 13.7) | 0.07 | 11.2 (8.8, 13.7) | 11.3 (8.8, 13.8) | <0.01 |
| Input & output (ml/kg) | 5.5 (3.3, 8.6) | 8.0 (4.4, 14.4) | 0.39 | 6.4 (3.8, 9.8) | 6.4 (3.6, 10.8) | 0.03 |
| Opioid in OR & RR (mg/kg) | 4.6 (0.4, 7.5) | 2.8 (0.0, 5.0) | 0.38 | 3.1 (0, 5.4) | 2.8 (0, 5.0) | 0.02 |
| Opioid after RR (mg/kg) | 0 (0.0, 43.3) | 35.7 (0.0, 175.4) | 0.38 | 0.0 (0, 90.2) | 0.0 (0, 111.3) | 0.04 |
