# Supplementary material for: Effect of the preoperative physical status on postoperative nausea and vomiting risk: a matched cohort study
Source: Perioper Med (Lond). 2022 Sep 6;11:31. doi: 10.1186/s13741-022-00264-1 (PMC9446728; doi:10.1186/s13741-022-00264-1)
Supplement: Supplementary file 3 — Additional file 3: Supporting Information Table 3. Characteristics and perioperative data before and after propensity score matching of ASA-PS 2 and 3 patients who underwent procedures under anesthesia except for local anesthesia in the sensitivity analysis. Values are number (percentages) or median (interquartile ranges). ASA, American Society of Anesthesiologists; PS, physical status; GY, gynecology; ENT; otorhinolaryngology; NPO, nothing by mouth; OR, operation room; RR, recovery room; ASD, absolute standardised difference. [file 13741_2022_264_MOESM3_ESM.docx]

**Supporting Information Table 3** Characteristics and perioperative data before and after propensity score matching of ASA-PS 2 and 3 patients who underwent procedures under anesthesia except for local anesthesia. Values are number (percentages) or median (interquartile ranges). ASA, American Society of Anesthesiologists; PS, physical status; GY, gynecology; ENT; otorhinolaryngology; NPO, nothing by mouth; OR, operation room; RR, recovery room; ASD, absolute standardised difference

|  | Before matching | | | After matching | | |
| --- | --- | --- | --- | --- | --- | --- |
|  | ASA PS 2  (n=106887) | ASA PS 3  (n=25015) | ASD | ASA PS 2  (n=23896) | ASA PS 3  (n=23896) | ASD |
| Young age (<50) | 50941 (47.7) | 3057 (12.2) | 1.08 | 3179 (13.3) | 3055 (12.8) | 0.02 |
| Female | 49833 (46.6) | 11705 (46.8) | <0.01 | 11292 (47.3) | 11218 (46.9) | 0.01 |
| Obesity | 11145 (10.4) | 2278 (9.1) | 0.05 | 2304 (9.6) | 2177 (9.1) | <0.01 |
| Smoking | 26510 (24.8) | 3410 (13.6) | 0.33 | 3342 (14.0) | 3361 (14.1) | 0.01 |
| Menstruation | 67 (0.1) | 6 (0.0) | 0.03 | 8 (0.0) | 6 (0.0) | <0.01 |
| Levin tube | 1606 (1.5) | 930 (3.7) | 0.12 | 787 (3.3) | 786 (3.3) | 0.01 |
| General Anesthesia | 88576 (82.9) | 22523 (90.0) | 0.24 | 21354 (89.4) | 21404 (89.6) | 0.01 |
| Inhalation Anesthetics | 82302 (77.0) | 20530 (82.1) | 0.13 | 19491 (81.6) | 19555 (81.8) | <0.01 |
| N2O | 10331 (9.7) | 1539 (6.2) | 0.15 | 1586 (6.6) | 1525 (6.4) | 0.01 |
| Remifentanil | 59094 (55.3) | 18688 (74.7) | 0.45 | 17507 (73.3) | 17597 (73.6) | <0.01 |
| Steroid | 4635 (4.3) | 1226 (4.9) | 0.03 | 1103 (4.6) | 1119 (4.7) | 0.01 |
| Neostigmine | 20024 (18.7) | 9173 (36.7) | 0.37 | 8162 (34.2) | 8285 (34.7) | <0.01 |
| Anticholinergics | 87778 (82.1) | 21514 (86.0) | 0.11 | 20489 (85.7) | 20480 (85.7) | <0.01 |
| Antiemetics | 82383 (77.1) | 21521 (86.0) | 0.26 | 20561 (86.0) | 20432 (85.5) | 0.01 |
| Laparoscopic surgery | 22724 (21.3) | 4571 (18.3) | 0.08 | 4351 (18.2) | 4413 (18.5) | 0.01 |
| Abdominal surgery | 20223 (18.9) | 5347 (21.4) | 0.06 | 4859 (20.3) | 5001 (20.9) | 0.01 |
| GY surgery | 10248 (9.6) | 783 (3.1) | 0.37 | 814 (3.4) | 772 (3.2) | 0.01 |
| EYE surgery | 1276 (1.2) | 277 (1.1) | 0.01 | 270 (1.1) | 276 (1.2) | <0.01 |
| ENT surgery | 11653 (10.9) | 1393 (5.6) | 0.23 | 1332 (5.6) | 1393 (5.8) | <0.01 |
| Head & neck surgery | 5322 (5.0) | 836 (3.3) | 0.09 | 882 (3.7) | 832 (3.5) | <0.01 |
| Anesthesia time (hour) | 100.0 (65.0, 150) | 125.0 (80.0, 185.0) | 0.29 | 120.0 (75, 185) | 120.0 (80, 180) | 0.01 |
| Recovery room time (hour) | 31.0 (25.0, 36) | 35.0 (24.0, 39.0) | 0.06 | 35.0 (28, 35) | 35.0 (24, 40) | 0.02 |
| NPO time (hour) | 11.2 (8.9, 13.8) | 11.3 (8.8, 13.7) | 0.02 | 11.2 (8.8, 13.6) | 11.2 (8.8, 13.7) | <0.01 |
| Input & output (ml/kg) | 5.7 (3.4, 9.4) | 8.0 (4.4, 14.4) | 0.32 | 7.3 (4.2, 12.4) | 7.7 (4.3, 13.6) | 0.01 |
| Opioid in OR & RR (mg/kg) | 4.0 (0.2, 6) | 2.8 (0.0, 5.0) | 0.31 | 3.2 (0.0, 5.1) | 2.8 (0.0, 5) | 0.03 |
| Opioid after RR (mg/kg) | 0.0 (0.0, 84.7) | 35.7 (0.0, 175.4) | 0.24 | 0.0 (0, 162.0) | 31.6 (0, 166.7) | 0.01 |
